# Evaluations of the curative efficacy of *G. fruticosus* solvent extracts in experimentally induced nephrolithiatic Wistar male rats

**DOI:** 10.1186/s12906-021-03320-3

**Published:** 2021-05-19

**Authors:** Tilahun Alelign, Tesfaye Sisay Tessema, Asfaw Debella, Beyene Petros

**Affiliations:** 1 Department of Microbial, Cellular, and Molecular Biology, College of Natural and Computational Sciences Addis Ababa University, P.O. Box 1176, Addis Ababa, Ethiopia; 2 Department of Biology, Debre Birhan University, P.O. Box 445, Debre Birhan, Ethiopia; 3 Institute of Biotechnology, Addis Ababa University, P.O. Box 1176, Addis Ababa, Ethiopia; 4 Ethiopian Public Health Institute, Traditional and Modern Medicine Directorate, Addis Ababa, Ethiopia

**Keywords:** Efficacy, *Gomphocarpus fruticosus*, In vivo, Urolithiasis

## Abstract

**Background:**

In Ethiopian folk medicine, there is a claim that medicinal plants can treat urolithiasis although there is insufficient scientific evidence. The objective of this study was to evaluate the curative efficacy of *Gomphocarpus fruticosus* extracts in experimentally induced nephrolithiatic rats.

**Methods:**

Urolithiasis was induced in male Wistar rats by feeding ethylene glycol in drinking water for 28 days. The curative effects were evaluated after oral administrations of 200 mg/kg of the extracts from 15 to 28 days. Urine samples were collected 1 day before sacrificing the rats. Blood, liver and kidney samples were gathered under anaesthetic condition at day 28. Crystals in the urine were also analyzed by light microscopy.

**Results:**

*G. fruticosus* EtOAc extract reduced significantly the level of sodium (*P* < 0.001), whereas it was significantly elevated the levels of magnesium and citrate (*P* < 0.01) compared to lithiatic control. *G. fruticosus* BuOH extract lowered the levels of potassium (*P* < 0.01), calcium and phosphate in urolithiatic rats. It was also observed that *G. fruticosus* EtOAc extract decreased the level of oxalate in the urine (*P* < 0.001), whereas it was increased the levels of magnesium (*P* < 0.05) and citrate (*P* < 0.01) in serum analysis after exposure to BuOH extract. In the kidneys, CaOx crystal deposits were reduced significantly by *G. fruticosus* EtOAc extract (*P* < 0.01).

**Conclusion:**

It has been noted that *G. fruticosus* EtOAc extract was potent in treating urolithiasis. However, further study is required to assess the efficacy of the active compounds against urolithiasis.

## Background

Urolithiasis (stones in the urinary tract) affects about 12% of the world population at some stage in their lifetime [1]. Other studies reported that the worldwide prevalence of kidney stone ranges between 2 to 20% [2, 3]. Urolithiasis affects all ages, sexes and races [4, 5], but it occurs more frequently in men than in women within the age 20 to 49 years [6]. The pathogenesis of kidney stones remain incompletely understood [7] and its treatment remains a challenge, although there are modern treatment modalities including ESWL [8]. The sequence of events that trigger stone formation includes nucleation, growth, aggregation, and retention of crystals within the kidneys [9, 10].

Renal stone formation requires persistent retention of crystals in the kidneys after the completion of the crystallization process [11, 12]. The interaction of COM crystals with a surface of renal epithelial cells is a critical initiating event in nephrolithiasis [13]. Crystal-cell interactions result in the movement of the crystals from the basolateral side of the cells to the basement membrane [14]. A study on animal models also revealed that the administration of high concentrations of CaOx crystals or oxalate ions appears to be toxic, causing renal tubular cell damage [7]. Exposure to higher levels of oxalate or CaOx crystals induce epithelial cell injury, which is a predisposing factor for the subsequent stone formation [15, 16].

An imbalance between urinary stone inhibitors and promoters has been suggested to be the cause of stone formation [17]. Promoters facilitate stone formation [18], but inhibitors decrease the initiation of supersaturation, nucleation, crystal growth and rate of aggregation [17]. However, inhibitors do work equally for everyone; therefore, some people form stones; while others do not [19]. Among recurrent stone formers, urinary oxalate excretion was higher, whereas citrate excretion was lower A study also indicated that oxalate can facilitate chloride, sodium and water re-absorption in the proximal tubules and activate multiple signaling pathways in renal epithelial cells [20].

Despite considerable improvements in medical therapy such as the utility of extracorporeal shock wave lithotripsy (ESWL), there is no satisfactory drug to treat renal calculi [21]. ESWL treatment is associated with acute renal injury due to the traumatic effect of the Shock wave, and infections after treatment [22]. It is also results in incomplete stone clearance, and unable to avoid new stone formations [23], or stone recurrences often up to 60% [24, 25]. After the first episode of a stone, the recurrence rate is more than 50% within 10-years [26, 27]. Therefore, kidney stone recurrence is a major risk, which is considered as the un-met clinical challenge in the area of urology [28]. Stone forming patients are prone to its recurrences after the first surgical therapy [29]. Even though the chemical compositions of a stone influence the choice of intervention [30], open surgery remain as the mainstay of treatment [31]. Some medicinal plants studied so far against urolithiasis are indicated in Table 1.

**Table 1:** Medicinal plants possessing anti-urolithiatic effects in previous studies

Plants name (Family)	Parts used	Study Model	Results/Effects	References
*Jasminum auriculatum* (Oleaceae)	Leaves	Male rats	Curative and preventive	[32, 33]
	Flowers	Male rats	Curative and preventive	[32]
*Sesbania grandiflora* (Fabaceae)	Leaves	Male rats	Antiurolithiatic effect	[34]
*Mimusops elengi* (Sapotaceae)	Bark	Male rats	Curative and preventive	[35]
*Hibiscus sabdariffa* linn. (Malvaceae)	Leaves	Male rats	Curative effect	[36]
*Moringa oleifera* Lam. (Moringaceae)	Root-wood (barks removed)	Male rats	Curative and preventive	[37, 38]
*Hygrophila spinosa* (Acanthaceae)	Whole plant	Male rats	Curative and preventive	[39]
*Chenopodium album* (Chenopodiaceae)	Leaves	Male rats	Stone inhibitory and dissolution effect	[40]

In Ethiopia, most patients rely on traditional medicinal plants as an alternative therapy for various diseases including urolithiasis. Medicinal plants are affordable, accessible, effective, with less side effects and being better compatible with the human body [41], compared to conventional drugs [42]. In the present study, *G. fruticosus* (L.) Ait.f were selected to examine their effects on experimentally induced urolithiasis. A thorough literature survey was carried out to ascertain that none of the selected medicinal plant parts have been studied so far on anti-urolithiatic activities. To date, there is also insufficiency of scientific evidence reported on the antiurolithiatic activity of this medicinal plant. Therefore, the objective of the study was to evaluate the curative efficacy of medicinal plants in experimentally induced nephrolithiatic male rats.

## Materials and methods

### Medicinal plant material identification and collection

*Gomphocarpus fruticosus* leaves were collected during its flowering or fruiting times. The *G. fruticosus* (L.) Ait.f, known as Tifriena (local name), was collected at Bole Bulbula around “93 Mazoria” Addis Ababa during October 2018. *G. fruticosus* L.Ait.f (Asclepiadaceae) (reported by key informant) was claimed for urolithiasis treatment. The plant specimens were submitted to the National Herbarium, Department of Plant Biology and Biodiversity Management, Addis Ababa University (AAU) for taxonomic authentication (TA238) and the corresponding collection number was given. The specimens were deposited in the National Herbarium of AAU for future references.

### Preparation of the crude extract

The plant materials were cleaned thoroughly with tap water to remove contaminants, and dried in shed at room temperature from 2 to 3 weeks in the Biomedical Sciences laboratory, AAU. The dried plant parts were finely powdered using a kitchen grinder (mortar and pestle, sized about 9 in. in diameter). The powders were put through a sieve of 3 mesh sizes so as to filter a gross solid matter.

The extracts were prepared using a procedure similar to that often used by traditional healer’s or patients, with some minor modifications. The plant powders were soaked in distilled water, which was placed on a shaker for 72 h. The mixture was filtered through cotton/gauze, then through Whatman filter paper number 1 (pore size: 11 μm) to remove fine solid plant particles or insoluble constituents. The entire extracts were concentrated to dryness using lyophilizer machine by removing of distilled water under reduced pressure. Then, the semi-solid concentrates poured into a glass petri-plates and allowed to completely dry in water bath adjusted to 45 °C. The final dried extracts were collected and stored in labeled sterile bottles covered with tightly stopper and kept in freezer on -20 °C until used in the experiments.

### Preparations of successive solvent extractions

*Gomphocarpus fruticosus* mother extract (aqueous) was partitioned with solvents of increasing polarity from non-polar to polar solvents ensuring that a wider polarity range of compounds could be extracted. Successive extraction was sequentially started with petroleum ether, followed by chloroform, ethyl acetate, butanol and water, in which these solvents were removed and concentrated under rotary vacuum evaporator. The plant materials macerated in distilled water were lyophilized under the freeze dryer to obtain the powdered residues. The dried extracts were capped tightly with stoppered and stored in glass bottles, and kept in a refrigerator at -20 °C until used for the experiment.

### Chemicals and reagents/standard drugs

The chemicals and reagents or kits used were analytical grade purchased from various sources. The procurement of petroleum ether, chloroform, ethyl acetate, butanol and ethanol were from Wisteam PLC (Addis Ababa, Ethiopia), potassium dihydrogen phosphate (anhydrous), sodium phosphate (dibasic anhydrous extra pure), Tris-HCl buffer (C_4_H_11_NO_3_), and sodium chloride (NaCl) were purchased from the Micron International Trading House PLC (Addis Ababa Ethiopia). Similarly, isoflurane, and formaldehyde were purchased from Neway Chemicals PLC (Addis Ababa, Ethiopia). Ethylene glycol (EG) and ammonium chloride (NH_4_Cl) were purchased from Pharma PLC (Addis Ababa, Ethiopia). EDTA tubes, serum separator tubes, capillary tubes and surgical blades were purchased from Micro Pharma PLC (Addis Ababa, Ethiopia). Furthermore, kits for liver and kidney function tests were purchased from a Roshi PLC (London, England). The oxalate (oxalic acid) colorimetric assay kits and citrate colorimetric/fluorometric assay kits were purchased from BioVision Incorporated (Milpitas, USA). Potassium citrate powder was obtained from the Black Lion Referral Hospital (Addis Ababa, Ethiopia). Cystone (polyherbal formulation) was purchased from Mumbi, India. Ascorbic acid, which was purchased from Micron International Trading House PLC (Addis Ababa, Ethiopia).

### Solubilization of the plant extract and the standard drugs

The test extracts/drugs of different concentrations were dissolved using appropriate vehicles, which were distilled water and 3% Tween 80. All plant extracts and potassium citrate were dissolved in distilled water at different concentrations. The procedure used to dissolve Cystone was similar to the methods of Phatak and Hendre [43] and Garimella et al. [44] with some modifications. Cystone tablets were powdered using a Mortar and Pestle (size: 0.23 m), and dissolved (suspended) in distilled water (900 μl) and 100 μl of 3% Tween 80 (that is, 750 mg/ml). Then, they were kept for 3 h to dissolve, centrifuged at 1000 rpm for 5 min, and filtered through 0.22 mm pore size filter paper. The clear supernatant was collected and used for calcium oxalate nucleation, and aggregation assays. Extracts were collected using Falcon tubes (45 ml), air tighten and stored in the refrigerator until experimental use. The test extracts and standard drugs were prepared daily and shortly prior to testing administrations at dose 200 mg/kg of extracts, 750 mg/kg of Cystone, and 2.5 g/kg of potassium citrate. The test substance dosing volume was 2 ml/100 g of body weight. Deionized water was used to prepare all extracts/drugs and these were prepared daily before testing administrations.

### In vivo urolithiatic pharmacological investigations

#### Target animals

Healthy adult male albino Wistar rats of the same age-group between 8 to 10 weeks and weighing (220-280 g) were used, purchased from the Ethiopian Public Health Institute (EPHI), and breed at the Biomedical Sciences animal laboratory, AAU. The experiment was conducted at the Biomedical Sciences Laboratory of AAU and EPHI, in accordance with internationally accepted standard guidelines for the use of animals in scientific research. Prior to starting the experiment, rats were acclimatized to standard laboratory conditions (6 rats per polypropylene cages) for 7 days. They were kept under a controlled environment of temperature (27 ± 2 °C), relative humidity (55 ± 5%), and light (12 h light/dark cycles). These rats were fed with regular pellets (standard diet) and allowed for free drinking water (ad libitum) for 28 days.

#### Urolithiasis induction

Kidney stones were induced using ethylene glycol (EG) along with ammonium chloride (NH_4_Cl) administered in the rats’ drinking water. In this hyperoxaluria model, 1% (w/v) NH_4_Cl was given with 0.75% (v/v) EG for the first 5 days to accelerate lithiasis, following this the water supply was switched to 0.75% EG alone for the next 25 days [45–47]. Exposure of these dose levels were sufficiently tolerable in animal studies [48]. EG administrations result in hyperoxaluria, which in turn leads to CaOx deposition in the kidneys [49]. The experimental rats assigned as stone curative groups were receiving stone inducing treatment for 28 days.

#### Curative effects of urolithiasis

In the curative treatment (dissolution), a total of 54 albino Wistar male rats were divided randomly into 9 groups comprising 6 individuals per group with matching body weights. In curative treatment, the disease was induced priory from day 1 to 14. Then, each of the extracts/drugs was administered orally from day 15 to 28 concurrently with disease induction (EG) protocols to determine curative effects [50, 51]. At the end of 28th days, rats were sacrificed for biochemical and histopathological studies. The experimental design were assigned as Group I (Normal control), Group II (Lithiatic control), Group III (K-Cit), Group IV (Cystone), Group V (*G. fruticosus* PET extract), Group VI (*G. fruticosus* Chl. extract), Group VII (*G. fruticosus* EtOAc extract), Group VIII (*G. fruticosus* BuOH extract), and Group IX (*G. fruticosus* aq. fraction). Other investigators were also used potassium citrate (2.5 g/kg) as a positive control [52, 53]. The dosing volume was 2 ml/100 g of body weight. The control group received distilled water once daily throughout the experiment. At the end of 28th days, rats were sacrificed for biochemical and histopathological studies.

#### Urine collection and microscopic analysis

Urine, serum and histological profiles are indicators of stone formation as well as recurrences. The abundance and morphology of calcium oxalate crystals formed under in vivo stone inductions were examined using a light microscope (Wagtech Thatcham Berkshire RG194QD, United Kingdom, 40x magnification).

Rats were placed in separate metabolic cages and subjected to 24 h urine collection at day 28th for curative test [50]. In crystalluria analysis, about 3 ml of the fresh urine samples collected were put in a glass tube and centrifuged at 3000 rpm for 10 min to remove debris, and supernatants were discarded. These urine sediments were used to determine CaOx crystal formation. About 10 μl of the vortexed sediments were placed onto a microscope glass slide (covered with a cover slip), and examined for CaOx crystals considering its number and size under a light microscope (40x). The crystals formed from chemicals in the urine were carefully examined from other urine artifacts. The photographs of microscopic observations were taken using a digital Camera (Sony Cyber-shot DSC-W180 10.1MP with 3x optical zoom, New Jersey, USA) manually mounted on top of it.

#### Urine biochemical analysis

At the end of the respective treatment periods, the animals were individually housed in metabolic cages, and 24 h urine (acidified and non-acidified) samples were collected. Urine was acidified with 1 ml of 6 N HCL (hydrochloric acid) and stored at 4 °C for 5 days. Then, these were centrifuged at 3000 rpm for 10 min (REMI, R24), and the supernatant of acidified urine were used to estimate excretions such as oxalate, calcium, magnesium and phosphate contents. In non-acidified urine sample contents such as citrate, creatinine and uric acid, and total protein concentrations were analyzed using commercially available diagnostic kits by the Automated clinical chemistry analyzer. Both oxalate and citrate concentrations were analyzed using multi-well spectrophotometer (ELISA reader) as per the manual provided with kits. For the purpose of quantifying, a calibration curve was prepared using Oxalate and Citrate kits as standard.

#### Serum collection and analysis

After the end of the experimental period (day 28th), rats were anesthetized using Isoflurane and 3 ml blood samples were collected from the retro-orbital vein by capillary puncturing. Serum was separated after centrifuged at 3000 rpm, 20 °C for 15 min. The collected serum was investigated for biochemical parameters like creatinine, blood urea nitrogen, uric acid, urea, calcium, magnesium, and phosphate by Clinical Chemistry Auto-analyzer (Cobas 6000 analyzer, Germany) with the respective diagnostic kits. The oxalate and citrate concentrations were determined using multi-well spectrophotometer (ELISA reader) as per the manual provided with kits. For the purpose of quantifying, a calibration curve was prepared using Oxalate and Citrate kits as standard.

#### Kidney homogenate analysis

At the end of the experimental period (day 28th), rats were sacrificed under Isoflurane anesthesia, followed by cervical dislocation. Then, the abdomen was opened and both kidneys collected from each rat. The isolated kidneys were carefully removed, and cleaned (washed/rinsed) from extraneous tissues with an ice cold physiological saline solution (0.15 M NaCl). The left kidneys from each animal was preserved in 10% buffered neutral formalin and used for histological studies. The right kidneys were sliced into two equal halves using a blade, and one-half dried at 80 °C in a hot air oven. The method used was similar to Ashok et al. [35] with some modifications, in which a fixed weight of 200 mg (29%) of the total kidney’s mean weight (0.67 g) was further heated separately in 10 ml of 1 N hydrochloric acid (1% HCl), which was placed in a boiled water bath of 100 °C for 30 min. Then, it was finely chopped into pieces using a blade, crashed by pestle and mortar, and further homogenized for 10 min using Ultra-Sonicator. The homogenate was centrifuged at 3000 rpm for 15 min, and the supernatant was collected using labeled Cryotubes. Finally, the supernatants were used for estimations of calcium, oxalate and phosphate contents with commercially available biochemical kits (BioVision PLC, USA), according to the manufacturer’s protocol [54, 55].

### Histopathological examinations

Histopathological examinations were done for kidney tissues of the experimental rats. All rats were sacrificed in a humane manner using Isoflurane anesthesia at the end of the 28th day (urolithiasis curative studies). The tissue pieces were taken from kidneys and analyzed for urolithiatic curative potentials of plant extracts. The tissues were fixed by 10% buffered neutral formalin solution, and subsequently embedded in paraffin wax. The sections (5 μm thick) were cut using Rotary Microtome 4060E (Germany), and mounted on glass slides and stained with Hematoxylin and Eosin to study the histopathological changes [34]. All fields of the tissue morphology were examined under a light microscope (100x magnification) (Wagtech Thatcham, Berkshire, RG19 4QD, United Kingdom), and the photomicrographs were captured using a digital Camera manually mounted it (Sony Cyber-shot DSC*-*W180 10.1MP with 3x optical Zoom, New Jersey, USA) for further references.

### Calcium oxalate crystal depositions

In the kidneys, crystal depositions were determined using semi-quantitative assays, which is a microscopic scoring method [46, 56]. The microscope filar micrometer (0.1 mm) eyepiece (10x, Wide field of 23.3 mm, Olympus Optics OSM 212422, made in Japan) was used. That is, crystal deposits in the kidney tissues were counted using a light microscope. The severity grades of crystal deposits were assigned as 0 = < 1 crystal (no crystal deposition), 1 = 1–10, 2 = 11–30, 3 = 31–50, 4 = 51–75 and 5= > 75 crystal counts, taking the mean values [46].

In counting CaOx crystal deposits, a sagittal section of each renal specimen was divided into four equal sized regions by two virtual lines, and readings of an average of 4 microscopic fields were reported [57, 58]. A field of 100x was then randomly selected from each region and CaOx deposits were counted. The image of one of the four regions under a light microscope was randomly captured [58] using a digital camera manually mounted on top of the Microscope.

### Statistical analysis

The data were analyzed using Graph Pad Prism version 6 Software (Graph Pad Software, San Diego, CA, USA). One-way analysis of variance (ANOVA) followed by post-hoc Dunnett’s test comparisons were performed when necessary to compare between treated and untreated groups. The data values were expressed as mean ± standard deviation (SD). Values of *P* < 0.05 was considered statistically significant.

## Results

### Curative efficacy of *G. fruticosus* extracts

In the curative (therapeutic) studies, the selected extracts dose was 200 mg/kg body weight of rats, which was one-tenth of the maximum tolerated dose 2000 mg/kg b.w [32]. This was chosen based on prior acute and/or sub-acute toxicity studies revealing its safety up to dose 2000 mg/kg. The induction of kidney stones by the administration of 0.75% EG combined with ammonium chloride (1%) in drinking water was confirmed in Wistar male rats.

### Urine (24 h) photomicroscopic analysis

**Fig. 1:**
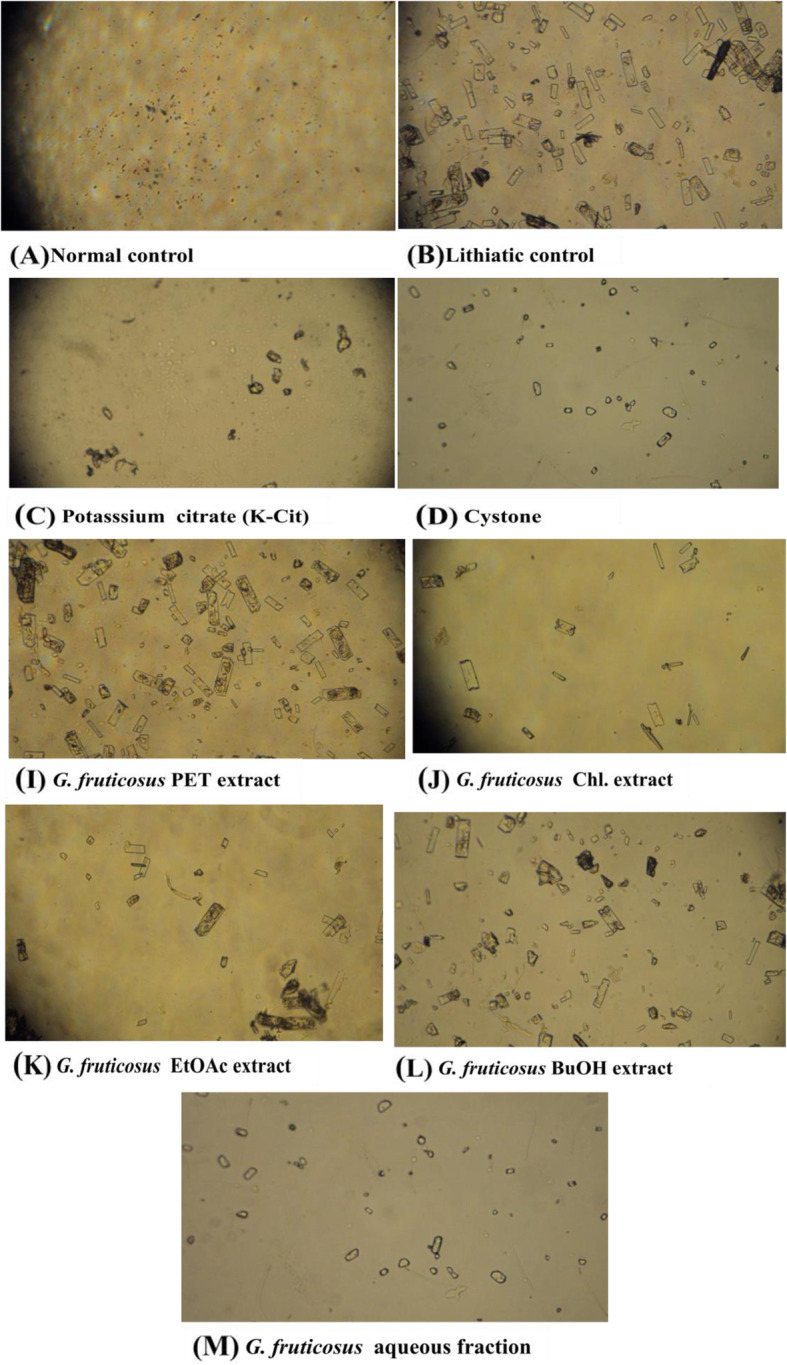
Urine photomicrographs of CaOx crystals following Curative treatment at 200 mg/kg extracts dose. The calcium oxalate crystal morphology and number viewed under a light microscope (40x), in morning urine from male Wistar rats **a** Normal control/vehicle; **b** Lithiatic control; treatment with **c** Potassium citrate (K-Cit); **d** Cystone; **i** *G. fruticosus* PET extract; **j** *G. fruticosus* Chl. extract; **k** *G. fruticosus* EtOAc extract; **l** *G. fruticosus* BuOH extract; and **m** *G. fruticosus* aqueous fraction

The analysis of urine samples showed variations in crystal density and size. Among the *G. fruticosus* successive solvent extracts, *G. fruticosus* EtOAc extracts (Fig. 1k) and *G. fruticosus* BuOH extracts (Fig. 1l) also reduced crystal numbers and sizes (Fig. 1).

### Urine and serum analysis for electrolytes and renal function test

In experimental rats, *G. fruticosus* EtOAc extract was reduced the level of sodium significantly compared to lithiatic control (*P* < 0.001). The level of potassium was reduced by *G. fruticosus* BuOH extract (*P* < 0.010). Treatment with the aqueous extract of EtOAc and BuOH extracts of *G. fruticosus* showed significant reductions in urinary protein excretions (*P* < 0.011) in relation to lithiatic control. Sodium concentration was also reduced very significantly with the treatment of EtOAc extract of *G. fruticosus* (*P* < 0.001). Treatment by the extract of *G. fruticosus* BuOH lowered the level of uric acid compared to lithiatic group (*P* < 0.050). However, lithogenic induction may cause impairments of renal function as evidenced by raising the levels of creatinine, proteins and uric acids. In the serum analysis, the level of potassium reduced by *G. fruticosus* EtOAc extract in comparison to lithiatic control (*P* < 0.010). It was also found that the effects of *G. fruticosus* BuOH extract on the level of chloride was close to the normal control. The level of creatinine raised by *G. fruticosus* crude extract and PET extract (*P* < 0.01) compared to the normal control (Table 2).

**Table 2:** Urinary (24 h) excretions and serum analysis of kidney stone-forming electrolytes and kidney function markers in experimental male Wistar rats treated with selected plant extracts at 200 mg/kg dose on 28th day post-treatment. The mixed extract = the combination of *A. aspera*, *S. punctata* and *R. abyssinicus* extracts (1:1:1 ratio), Aqueous (aq.), Ethyl acetate (EtOAc), Butanol (BuOH), Chloroform (Chl), and Petroleum ether (PET). The data were presented the mean ± SD for 6 rats in each group (*n* = 6). Comparisons between means were made against Group I (vehicle control) and Group II (lithiatic control). *p < 0.05, **p < 0.01, ***p < 0.001 indicate significant changes in comparison with Group I (vehicle control), #*p* < 0.05, ##*p* < 0.01, ###*p* < 0.001 indicate a significant change in comparison with Group II (lithiatic control)

Groups	Treatments	Electrolytes (mg/dl)			Kidney Function Test (mg/dl)		
		Sodium	Potassium	Chloride	Creatinine	Total protein	Uric acid
***Urine***							
I.	Normal control (DH_2_O)	135 ± 33.85	25.33 ± 6.44	142.56 ± 17.71	1.11 ± 0.09	3.50 ± 0.74	2.14 ± 0.33
II.	Lithiatic control	213 ± 48.14	43.00 ± 11.91	189.30 ± 21.65	5.11 ± 2.53	12.13 ± 5.35	5.13 ± 1.71
III.	K-Cit (2.5 g/kg)	154 ± 32.32#	35.75 ± 7.03	145.20 ± 15.25#	2.51 ± 0.76#	7.66 ± 2.90*#	3.05 ± 0.98
IV.	Cystone (750 mg/kg)	163 ± 18.14#	38.03 ± 5.19	157.58 ± 16.51	3.13 ± 1.03*	8.50 ± 1.57**	3.12 ± 1.07
V.	*G. fruticosus* PET extracts	204 ± 34.05**	43.98 ± 7.11*	166.03 ± 15.12*	4.15 ± 0.33**	9.15 ± 1.87**	5.11 ± 0.33*
VI.	*G. fruticosus* Chl. extracts	213 ± 33.71**	39.83 ± 6.45	164.50 ± 15.73*	3.68 ± 0.66*	8.98 ± 3.32**	5.30 ± 1.20*
VII.	*G. fruticosus* EtOAc extra	128.5 ± 10.19###	31.08 ± 4.50	144.21 ± 20.06#	3.41 ± 0.47*	4.66 ± 1.09##	4.83 ± 0.71
VIII.	*G. fruticosus* BuOH extracts	133 ± 15.04##	21.41 ± 3.82##	143.78 ± 14.37#	3.36 ± 0.87*	4.97 ± 0.75##	3.91 ± 0. 67#
IX.	*G. fruticosus* aq. fraction	148 ± 17.03#	27.50 ± 6.73#	148.00 ± 18.00#	3.35 ± 0.12*	8.90 ± 2.15*#	3.79 ± 2.11
***Serum***							
I.	Normal control (DH_2_O)	157 ± 6.25	5.17 ± 1.25	82 ± 4.33	1.03 ± 0.05	3.50 ± 0.74	0.97 ± 0.08
II.	Lithiatic control	219 ± 13.24	9. 14 ± 2.31	113. ± 7.25	5.29 ± 0.89	13.19 ± 3.02	6.74 ± 2.34
III.	K-Cit (2.5 g/kg)	16*.5 ± 6.19#	7.3 ± 1.77	93 ± 6.19	2.68 ± 0.50#	5.63 ± 1.34##	2.50 ± 1.05##
IV.	Cystone (750 mg/kg)	175 ± 10.32#	6.1 ± 2.03	88 ± 6.22	3.19 ± 0.39	7.85 ± 2.55*#	4.21 ± 0.89#
V.	*G. fruticosus* PET extract	203 ± 17.16**	8.20 ± 2.13*	129 ± 6.51*	5.93 ± 0.16**	7.82 ± 1.20*#	4.18 ± 0.78*
VI.	*G. fruticosus* Chl. extract	193 ± 12.50*	9.50 ± 2.17*	122 ± 9.54*	4.03 ± 0.39*	6.93 ± 1.54*#	5.40 ± 1.33**
VII.	*G. fruticosus* EtOAc extract	169 ± 8.34#	4.85 ± 0.93##	94 ± 5.78	3.67 ± 0.04	5.04 ± 1.43##	2.76 ± 0.61##
VIII.	*G. fruticosus* BuOH extract	174 ± 11.23#	8.22 ± 2.07*	79 ± 4.13#	3.19 ± 0.07	7.98 ± 2.17*#	5.22 ± 1.27*
IX.	*G. fruticosus* aq. fraction	185 ± 8.37*	5.16 ± 1.64#	87 ± 4.84#	3.18 ± 0.60	6.23 ± 2.17*#	4.93 ± 0.44*

### Urine and serum analysis of crystal formation inhibitors and promoters

As indicated in Table 3, the level of magnesium was significantly elevated by supplementation of *G. fruticosus* EtOAc extract compared to lithiatic control (*P* < 0.010). Similar to K-Cit, the effects of *G. fruticosus* EtOAc extract increased the level of citrate significantly (*P* < 0.010). *G. fruticosus* BuOH extract lowered the elevated levels of calcium and phosphate significantly in the urine compared to the diseased (lithiatic) control (Group II rats) (*P* < 0.010). It was also observed that *G. fruticosus* EtOAc extract decreased the level of oxalate in the urine compared to lithiatic control (*P* < 0.001). In the serum analysis, the excretions of calcium, oxalate and phosphate were grossly increased in lithiatic induced male Wistar rats. The EtOAc extract of *G. fruticosus* increased the concentration of magnesium (*P* < 0.052). The serum level of citrate was increased after exposure to BuOH extract of *G. fruticosus* (*P* < 0.011), suggesting similar effects to potassium citrate (*P* < 0.001). *G. fruticosus* Chl. extract significantly reduced the citrate level compared to the normal control (*P* < 0.001) (Table 3).

**Table 3:** Effects of plant extracts at 200 mg/kg dose on changes in urinary excretion (24 h) and serum crystal inhibitors and promoters in male Wistar rats on the 28th day post-treatment. The mixed extract = the combination of *A. aspera*, *S. punctata* and *R. abyssinicus* extracts (1: 1:1 ratio). The data were presented as mean ± SD for 6 rats in each group (*n* = 6). Comparisons between means were made against Group I (vehicle control) and Group II (lithiatic control). *p < 0.05, **p < 0.01,***p < 0.001 indicate significant changes in comparison with Group-I (vehicle control), #*p* < 0.05, ##*p* < 0.01, ###*p* < 0.001 indicate significant changes in comparison with Group II (lithiatic control)

Groups	Treatments	Crystal inhibitors (mg/dl)		Crystal promoters (mg/dl)		
		Magnesium	Citrate	Calcium	Phosphate	Oxalate
***Urine***						
I.	Normal control (DH_2_O)	2.88 ± 0.63	3.46 ± 0.78	2.60 ± 0.48	5.35 ± 1.12	3.81 ± 0.43
II.	Lithiatic control	0.96 ± 0.14	1.83 ± 0.51	11.40 ± 3.14	9.71 ± 2.89	12.76 ± 3.62
II.	K-Cit (2.5 g/kg)	1.52 ± 0.42	4.32 ± 1.43##	6.28 ± 1.15*#	6.73 ± 1.34	7.53 ± 2.02*#
IV.	Cystone (750 mg/kg)	1.70 ± 0.34	2.21 ± 0.46	5.84 ± 1.23*#	5.31 ± 1.29#	8.29 ± 2.11**
V.	*G. fruticosus* PET extract	1.87 ± 0.65	1.98 ± 0.23*	8.11 ± 1.29*	7.04 ± 2.17	7.32 ± 0.91*#
VI.	*G. fruticosus* Chl. extract	1.57 ± 0.28	2.37 ± 0.04	7.07 ± 1.16*	8.3 ± 2.62*	7.35 ± 1.40*#
VII.	*G. fruticosus* EtOAc extract	3.30 ± 1.21##	3.02 ± 1.12##	6.24 ± 1.35#	5.12 ± 0.98#	4.25 ± 0.53###
VIII.	*G. fruticosus* BuOH extract	2.61 ± 0.74#	2.43 ± 0.91	4.17 ± 0.85##	4.10 ± 0.65##	4.39 ± 0.33##
IX.	*G. fruticosus* aq. fraction	2.50 ± 0.88#	2.95 ± 0.66#	5.16 ± 0.71#	5.41 ± 1.02#	6.51 ± 1.03*##
***Serum***						
I.	Normal control (DH_2_O)	3.07 ± 0.51	5.11 ± 1.33	3.17 ± 0.97	1.78 ± 0.64	1.24 ± 0.21
II.	Lithiatic control	0.98 ± 0.07	1.20 ± 0.07	11.6 ± 2.13	5.14 ± 1.45	11.32 ± 2.27
III.	K-Cit (2.5 g/kg)	1.72 ± 0.12	5.41 ± 1.13###	6.11 ± 1.75*#	3.61 ± 0.61*	4.06 ± 0.83*##
IV.	Cystone (750 mg/kg)	1.50 ± 0.30*	3.00 ± 0.32#	8.24 ± 2.88**	3.33 ± 1.14*	3.57 ± 0.67*###
V.	*G. fruticosus* PET extract	0.79 ± 0.08**	2.43 ± 0.79*#	7.44 ± 2.50**	6.78 ± 1.14*	6.75 ± 1.29**#
VI.	*G. fruticosus* Chl. extract	1.44 ± 0.19*	0.93 ± 1.19***	8.30 ± 2.11**	4.39 ± 0.87*	7.92 ± 2.31**
VII.	*G. fruticosus* EtOAc extract	2.47 ± 0.11#	1.68 ± 1.27**	5.25 ± 1.62#	3.06 ± 0.32*	4.72 ± 0.68**##
VIII.	*G. fruticosus* BuOH extract	1.04 ± 0.31**	4.58 ± 1.54##	5.14 ± 0.82#	4.48 ± 1.89*	5.86 ± 2.14**##
IX.	*G. fruticosus* aq. fraction	1.87 ± 0.15	2.50 ± 0.92*#	3.42 ± 0.84###	6.03 ± 1.57*	7.10 ± 2.05**

The serum level of alanine aminotransferase (ALT), and aspartate aminotransferase (AST) increased in the lithiatic control compared to the normal (healthy) control (*P* < 0.050). *G. fruticosus* PET extract increased serum AST level significantly (*P* < 0.001) compared to the healthy control. BuOH extract of *G. fruticosus* lowered significantly the level of AST similar to K-Cit (*P* < 0.001) compared to lithiatic control (Table 4).

**Table 4:** Effects of plant extracts on the serum enzyme activity in relation to kidney stone treatment at 200 mg/kg extract dose on the 28th day post-treatment. The mixed extract = the combination of *A. aspera*, *S. punctata* and *R. abyssinicus* extracts (1:1:1 ratio). Data were presented as mean ± SD for 6 rats in each group (*n* = 6). Comparisons between means were made against Group I (vehicle control) and Group II (lithiatic control). *p < 0.05, **p < 0.01,***p < 0.001 indicate significant changes in comparison with group I (vehicle control), #*p* < 0.05, ##*p* < 0.01, ###*p* < 0.001 indicate significant changes in comparison with group II (lithiatic control)

Groups	Treatments	Enzyme Activity	
		ALT (IU/L)	AST (IU/L)
**I.**	Normal control (DH_2_O)	68.4 ± 5.04	113.1 ± 6.53
**II.**	Lithiatic control	135.3 ± 14.13	211.0 ± 23.10
**III.**	K-Cit (2.5 g/kg)	78.7 ± 6.10##	126.7 ± 8.11###
**IV.**	Cystone (750 mg/kg)	67.8 ± 5.89##	161.3 ± 10.32*##
**V.**	*G. fruticosus* PET extract	123.1 ± 11.05***	225.1 ± 23.12***
**VI.**	*G. fruticosus* Chl. extract	115.8 ± 7.01***	208.7 ± 23.13***
**VII.**	*G. fruticosus* EtOAc extract	78.7 ± 3.85*###	219.4 ± 27.60***
**VIII.**	*G. fruticosus* BuOH extract	71.4 ± 5.16###	139.3 ± 9.54*###
**IX.**	*G. fruticosus* aq. fraction	69.2 ± 4.11###	120.6. ± 11.32**###

### Kidney homogenate analysis

The concentrations of calcium, phosphorus and oxalate levels were increased in the lithiatic group (Group II) compared to the normal control (Group I). All treatments showed significant reductions in the level of phosphate (*P* < 0.050), except treatment with BuOH and Chl. extracts of *G. fruticosus*. The administration of the aqueous mixed extract and *G. fruticosus* EtOAc extract reduced the level of oxalate significantly compared to the untreated lithiatic groups (*P* < 0.011) (Table 5).

**Table 5:** Effect of plant extracts on kidney homogenate constituents of phosphate, oxalate and calcium following the administrations of curative test on the 28th day at 200 mg/kg dose. The mixed extract = the combination of *A. aspera*, *S. punctata* and *R. abyssinicus* extracts (1: 1:1 ratio). Data were presented as mean ± SD for 6 rats in each group (*n* = 6). Comparisons between means were made against Group I (vehicle control) and Group II (lithiatic control). *p < 0.05, **p < 0.01,***p < 0.001 indicate significant changes in comparison with the group I (vehicle control), #*p* < 0.05, ##*p* < 0.01, ###*p* < 0.001 indicate significant changes in comparison with group II (lithiatic control)

Groups	Treatments	Kidney homogenates (mg/dl)		
		Phosphate	Oxalate	Calcium
I.	Normal control (DH_2_O)	3.84 ± 1.02	1.45 ± 0.30	2.12 ± 1.30
II.	Lithiatic control	9.44 ± 2.31	7.67 ± 2.25	9.50 ± 2.47
III.	K-Cit (2.5 g/kg)	5.13 ± 1.33#	3.33 ± 0.87*#	3.33 ± 2.24#
IV.	Cystone (750 mg/kg)	7.05 ± 2.00	5.27 ± 1.42**	4.22 ± 2.10#
V.	*G. fruticosus* PET extract	8.23 ± 2.70*	4.52 ± 0.56*#	5.03 ± 1.98
VI.	*G. fruticosus* Chl. extract	7.50 ± 2.08	4.09 ± 0.78*#	5.11 ± 1.69
VII.	*G. fruticosus* EtOAc extract	3.89 ± 0.42#	2.10 ± 0.87##	7.05 ± 2.56*
VIII.	*G. fruticosus* BuOH extract	6.11 ± 1.55	3.31 ± 0.32*#	7.21 ± 2.37*
IX.	*G. fruticosus* aq. fraction	4.52 ± 0.63#	5.43 ± 1.05**	5.53 ± 1.88*

### Evaluations of kidneys against CaOx crystal depositions

**Fig. 2:**
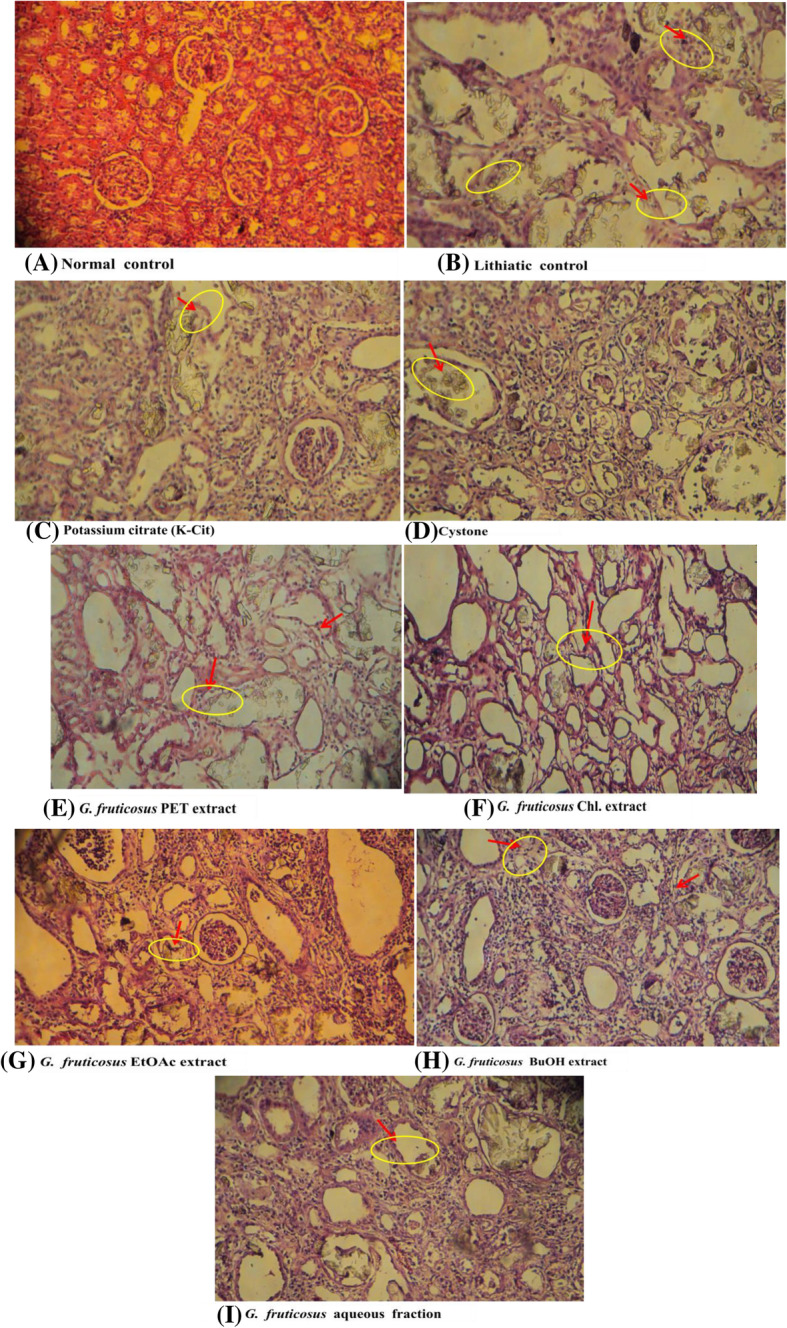
Representative photomicroscopic (100x) images of kidney sections of test male Wistar rats in plant extracts for anti-CaOx crystal deposition effect (dose of extracts 200 mg/kg). Histopathology of kidney tissues **a** Normal control/vehicle, **b** Lithiatic control, treatment with **c** K-Cit, **d** Cystone, **e** *G. fruticosus* PET extract, **f** *G. fruticosus* Chl. extract, **g** *G. fruticosus* EtOAc extract, **h** *G. fruticosus* BuOH extract, and **i** *G. fruticosus* aq. fraction. The mixed extracts (the combination of *A. aspera*, *S. punctata* and *R. abyssinicus* extracts in 1:1:1 ratio). Polymorphic irregular CaOx crystals in the renal tubules (yellow circle/arrows). Images were 5 μm thick paraffin sections with Hematoxylin-Eosin stain

Light microscopic examination for CaOx crystals in kidney histologic sections revealed CaOx crystals in the tubular lumen. The size and amount of CaOx deposits decreased among treated groups in comparison with lithiatic controls. The effect of *G. fruticosus* EtOAc extract (G), and its BuOH fraction (H) reduced the number of crystals compared to lithiatic control (B), but do not reduced for aqueous extracts of *G. fruticosus* (I) (Fig. 2).

### Effects of *G. fruticosus* extracts on CaOx deposition in the kidneys

**Fig. 3:**
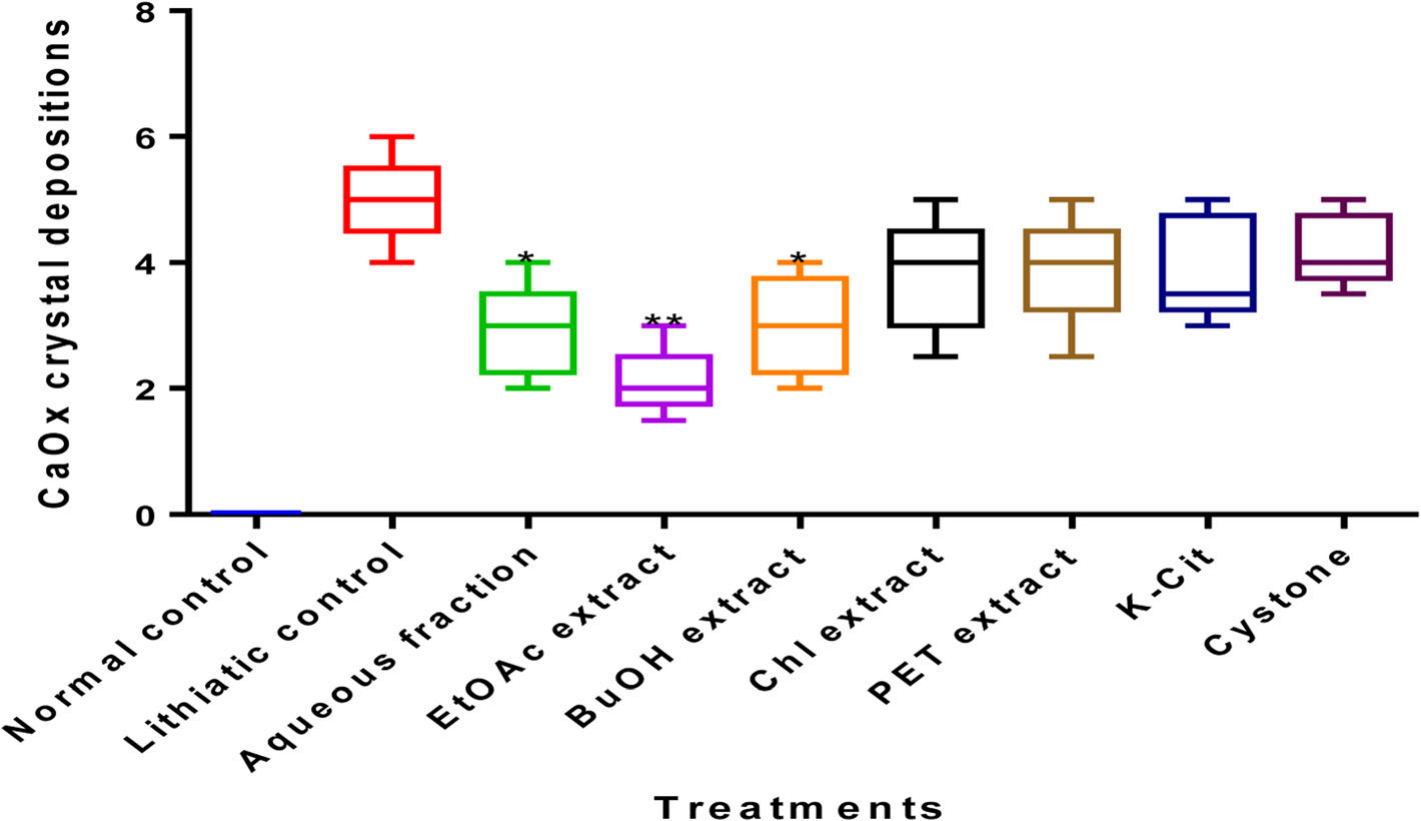
CaOx crystal deposition scores in the curative study after treatment with various successive extracts of *G. fruticosus* (dose of extracts:200 mg/kg). Data were expressed as mean ± SD of *n* = 6 rats per group. *p < 0.05, **p < 0.01, and ***p < 0.001 indicates a significant change in comparison with lithiatic control (hyperoxaluric group)

The number of crystal deposits was counted from the cortex, medulla and papilla by taking the mean of 4 microscopic fields. In comparison with lithogenic groups, CaOx crystal deposits in the kidneys were reduced significantly by *G. fruticosus* EtOAc extract (*p* < 0.010). The number of crystal deposits was counted via a sagittal section or longitudinal plane of each renal tissue specimen divided into 4 equal sized regions (two virtual lines) and taking the mean of all fields (Fig. 3). It has been noted that *G. fruticosus* EtOAc extract was the most potent agent in treating urolithiasis followed by BuOH extract.

## Discussion

The kidneys filter waste products from the blood and void them into the urine, this is not possible if waste materials do not dissolve completely in the urine, leading to kidney stone formation [59]. The biomineralization processes involve successive physicochemical changes such as super-saturation, nucleation, growth, aggregation and retention within renal tubules [60, 61]. Hyperoxaluria is the main risk factor for kidney stone formation than hypercalciuria [62].

Urinary excretions of oxalate, phosphate, and calcium were increased, while the levels of magnesium and citrate levels reduced in the urine compared to the treated group at dose 200 mg/kg b.w, which were similar to the studies reported by Lemann et al. [63] and Sathish et al. [39]. The increment of mineral constituents in the urine could be due to defective renal tubular re-absorptions. Similarly, electrolytes (sodium, potassium and chloride) are used to screen renal acidosis that facilitate renal stone formations [64]. In curative study, sodium level was reduced significantly by *G. fruticosus* EtOAc extract (*P* < 0.001). In contrast, previous studies reported that excessive excretions of sodium and chloride in the urine indicate the diuretic activity of the test extract [65, 66], with increasing urinary output, which inhibit stone developments [67].

Magnesium binds with oxalate ions to form soluble oxalate complexes in the urine, and decreases the oxalate availability, which binds with calcium leading to CaOx formation [68, 69]. Hence, it could be suggested that CaOx stones are most likely to be formed by people who are magnesium deficient. This was supported with the fact that *G. fruticosus* EtOAc extract elevated the levels of magnesium and citrate (*P* < 0.010) similar to K-Cit in the curative test. The EtOAc extract of *G. fruticosus* decreased the levels of oxalate in the urine (*P* < 0.001) compared to lithiatic control in the curative study. In the curative test, significant (*P* < 0.011) reductions in urinary levels of calcium and phosphate following *G. fruticosus* BuOH extract administration indicates the presence of CaOx inhibitory constituents, which interfere with crystal nucleation and aggregations. Moreover, an increase in calcium concentrations is a favoring environment for nucleation, and precipitation of calcium oxalate or calcium phosphate with subsequent crystal growth [32, 39]. Similarly, an increased urinary phosphate and oxalate excretions also provide a favorable environment for the formation of calcium phosphate, in turn, leading to calcium oxalate crystal depositions in the renal tubules [18, 70].

In curative study, *G. fruticosus* EtOAc extract (*P* < 0.010) reduced serum levels of potassium. In addition, the effects of *G. fruticosus* BuOH extract on the level of chloride was found to be close to the normal control during the curative study. The serum level of sodium was significantly reduced by *G. fruticosus* BuOH extracts (*P* < 0.050) compared to lithiatic control in the curative study. There was a decrease in serum oxalate excretions by EtOAc and BuOH extracts of *G. fruticosus* compared to lithiatic control. This might be either due to the inhibitions of oxalate formation, or interference with oxalate metabolism. Similarly, *G. fruticosus* EtOAc and BuOH extracts (*p* < 0.050) decreased calcium levels compared to lithiatic control in curative test. The ability of these extracts to alter calcium and oxalate excretions may be due to the disintegration of mucoproteins, which are promoters of crystallization as reported by Doddola et al. [34]. In the present study, the level of citrate in the serum was increased by *G. fruticosus* BuOH extracts (*P* < 0.011), suggesting a possible curative effect close to K-Cit treated group (*P* < 0.001). The presence of tannins and flavonoids can lead to the relaxation of smooth muscles of the urinary tract, which could facilitate the expulsion of kidney stones as studied in rats [54], which were confirmed their presence in the phytochemical analysis of *G. fruticosus* extracts in the present study.

In the present therapeutic study, it was also evidently proved that the *G. fruticosus* BuOH extracts lowered AST levels very significantly (*P* < 0.001), which was close to K-Cit compared to the lithiatic control in the curative studies. This can be attributed to the fact that abnormal levels of liver enzymes, particularly aminotransferases including alanine aminotransferase (ALT), and aspartate aminotransferase (AST) are prognostic features (indicators) of the damages of liver cells, and the cellular integrity of the kidneys [71, 72].

Experimentally induced CaOx crystal deposition in the kidneys is also associated with localized inflammation as evidenced by infltration of monocyte and macrophages to the site [73]. *G. fruticosus* EtOAc extract (*P* < 0.051) and its BuOH extract (*P* < 0.051) treated groups, the severity of CaOx crystal depositions reduced the kidneys compared to lithiatic control, which was similar to a study on the other herbal extract [74]. In curative study, EtOAc extract of *G. fruticosus* reduced the level of phosphate significantly (*P* < 0.051) compared to the lithiatic control. Tissue injury could be caused by exposures to phosphate and calcium phosphate crystals, leading to the generation of oxidative stress, lipid peroxidation and depletion of antioxidant enzymes [75]. Consequently, the renal epithelial injury promotes crystal retention, as epithelial injury exposes a variety of crystal adhesion molecules on epithelial surfaces and promotes stone formation [73, 76]. COM accumulates in the kidneys by attaching to tubular cell membranes, followed by internalization by endocytosis, leading into cell death [75]. The precipitation of oxalate as COM in the tubular lumen has been linked with renal toxicity and inflammation, damaging the structures of mitochondria, and inhibiting mitochondrial respiratory functions in proximal tubular cells, alter cellular permeability and leading to renal cell death [77].

A major limitation of the study is the inability to use a polarized light microscope that magnifies and better resolve in vivo crystallizations to differentiate between COM and COD crystals which would have improved data quality. The effects of test extracts on urinary diuretic activities were not assessed. Despite these limitations, the strength of the present study was the use of standard experimental designs and procedures to test the potential efficacy of the traditional medicinal plants used in the study.

## Conclusions

The present study has provided some evidences for the traditional use of *G. fruticosus* extracts in urolithiasis management. In vivo/animal tests confirmed the curative efficacies of *G. fruticosus* extracts*,* which were evidenced from biochemical and histopathological findings. The present findings demonstrated the CaOx curative efficacy of *G. fruticosus* EtOAc extract. Further research is necessary to identify the active compounds responsible for the treatment of urolithiasis.

## Data Availability

The data generated or analyzed during this study are included in the article.
